# The Influence of Radiological “Disappearing Lesions” on the Efficacy and Prognosis of Patients with Colorectal Liver Metastases Undergoing Conversion Therapy

**DOI:** 10.1155/2022/2200598

**Published:** 2022-02-22

**Authors:** Zhi-yang Song, Dong Yang, Yang Liu, Yong Cheng

**Affiliations:** ^1^Department of Gastrointestinal Surgery, The First Affiliated Hospital of Chongqing Medical University, Chongqing 400000, China; ^2^Radiology Department, The First Affiliated Hospital of Chongqing Medical University, Chongqing 400000, China

## Abstract

**Purpose:**

The purpose of the current study was to analyze the influence of radiological “disappearing liver metastasis” (DLM) on the efficacy and prognosis of patients with colorectal liver metastases (CRLM) undergoing conversion therapy.

**Methods:**

Patients with CRLM by the multidisciplinary team (MDT) of the First Affiliated Hospital of Chongqing Medical University were retrospectively enrolled from January 2014 to January 2021. The relationship between the occurrence and recurrence of DLM and different clinical factors was analyzed.

**Results:**

Thirty-five of the 113 patients (31.0%) with initially unresectable CRLM developed DLM, and of the 361 lesions, 177 disappeared (49.0%). Within 6 months, 6-12 months, and 12-24 months groups, the recurrence rate was 3.4%, 16.8%, and 34.8%, but there is no recurrence in after 24 months group. There was a statistical difference between chemotherapy alone and chemotherapy combined with the targeted therapy group on the occurrence of DLM (58.3% vs. 37.1%, *P* < 0.001). There were significant differences between <5 mm group and >10 mm group on occurrence of DLM（76.7% vs. 30.4%, *P* < 0.001) and between 5-10 mm group and >10 mm group also (70.0% vs. 30.4%, *P* < 0.001). Through univariate and multivariate analyses, it was concluded that age (*P* = 0.026, 95%CI = 3.690) and treatment regimens (*P* = 0.033, 95%CI = 2.703) had a significant influence on the progression-free survival (PFS) time of DLM.

**Conclusion:**

Younger patients, who use chemotherapy alone to achieve a therapeutic effect, might have better survival benefits when the lesions do not progress within 2 years after the appearance of DLMs.

## 1. Introduction

Colorectal cancer (CRC) is the third most common malignant tumor globally, and its incidence and mortality rank fifth in the world [1]. About 50% of the new cases of CRC develop liver metastases during their progression each year [1, 2]. The liver is the main target organ for CRC metastasis, and liver metastasis is the leading cause of poor efficacy, prognosis, and death in patients with CRC [3, 4]. Surgical resection of liver metastases is preferred in the current treatment options [5–7]. However, a part of patients with colorectal liver metastases (CRLM) are considered initially unresectable, in which conversion therapy plays a significant role in prolonging the overall survival of patients and reducing tumor recurrence [8].

Conversion therapy is expected to transform the unresectable CRLM into resectable status, including chemotherapy, targeted therapy, and radiofrequency ablation [9]. Previous studies have claimed that about 7-35% of patients who undergo conversion therapy have liver metastases disappear radiologically, namely, “disappearing liver metastases” (DLM) [10–12]. Studies have claimed that computed tomography (CT) and magnetic resonance imaging (MRI) are reliable imaging methods for the diagnosis of CRLM [2, 13]. There are still no consistent recommendations on treating the lesions [2, 14]. Some researchers suggest that CRLM patients with DLM still need local resection for their disappeared site. But surgical resection may lead to tremendous trauma and affect the later quality of life. However, some studies have claimed that if the DLM is left with regular follow-up, it is effective to undergo surgical treatment when the lesions recur. Some patients even get better survival benefits because of the appearance of DLM [2, 5–7, 15].

This study retrospectively analyzed the clinical data, follow-up time, and tumor changes recorded in imaging of single liver metastatic lesion of CRC with DLM after chemotherapy and/or targeted therapy and analyzed the correlation factors with the appearance and recurrence of DLM. The purpose of the current study was to analyze the influence of radiological DLM on the efficacy and prognosis of patients with CRLM undergoing conversion therapy.

## 2. Materials and Methods

### 2.1. Patients

**Figure 1:**
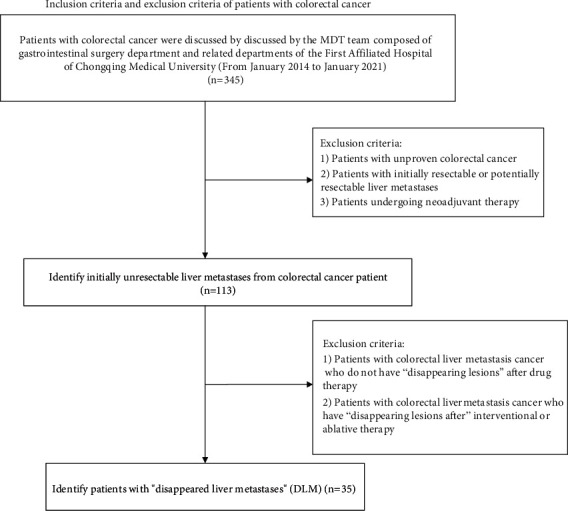
Inclusion criteria and exclusion criteria of patients with colorectal cancer. Abbreviations: DLM: disappearing liver metastasis.

Patients with CRC were enrolled from January 2014 to January 2021 who were discussed by the multidisciplinary team (MDT) composed of the gastrointestinal surgery department and related departments of the First Affiliated Hospital of Chongqing Medical University. This study was approved by the ethics committee (2021-521), and all patients signed the informed consent. The inclusion criteria were as follow: (1) pathologically confirmed CRC and (2) initially unresectable liver metastasis lesions. The exclusion criteria were as follow: (1) no pathologically confirmed CRC; (2) initially resectable or potentially resectable liver metastases; (3) undergoing neoadjuvant therapy; and (4) no DLM after conversion therapy or have DLM after interventional or ablative therapy (Figure 1).

The clinical data corresponding each liver metastasis was analyzed among the patients screened according to the inclusion and exclusion criteria. Some liver metastases in a few patients were too many to be accurately counted but isolated and dense, so the number of the lesions was set at 30, among which only the disappearing lesions and their corresponding clinical data were analyzed. In the statistics of the number of DLMs, some lesions were not included in the statistics because patients gave up treatment after the initial diagnosis of DLM.

### 2.2. Imaging

**Figure 2:**
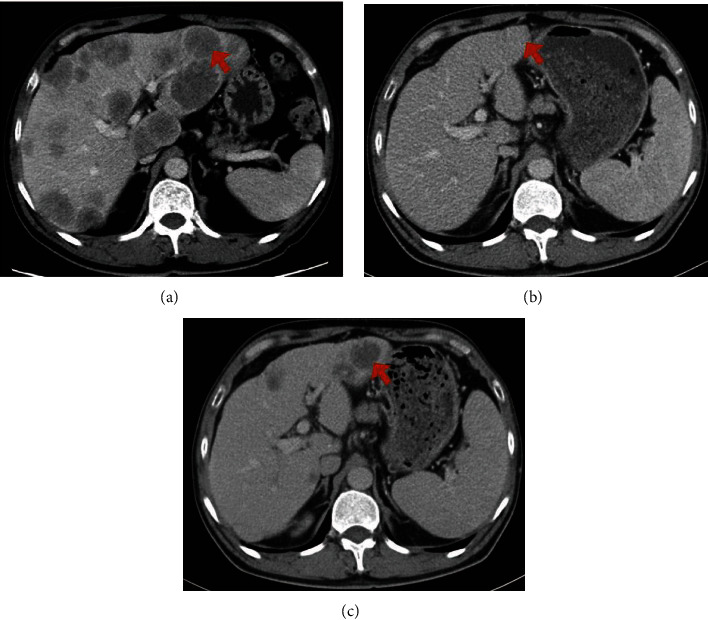
Examples of DLM on preoperative image in patients with CRLM. (a) Image showing the CRLM in the first diagnosis time (arrow). (b) Image showing DLM after conversion therapy (arrow). (c) Image showing recurrence of DLM with follow-up (arrow). Abbreviations: CRLM: colorectal liver metastases; DLM: disappearing liver metastasis; MRI: magnetic resonance imaging.

Many studies have shown that both computed tomography (CT) and magnetic resonance imaging (MRI) may be more than 90% sensitive for detecting liver lesions larger than 1 cm [16]. MRI detection of lesions smaller than 1 cm is more sensitive than CT (53% vs. 36%, respectively) [17, 18]. The imaging methods included multiphase enhanced CT scan and enhanced MRI involved in this study. There are specific manifestations of the DLM on imaging: CT findings were as follows: (a) the original low-density lesion disappeared completely, and/or (b) the lesion was not enhanced after contrast administration. MRI findings were as follows: (a) the abnormal signal of the original metastatic lesion disappeared completely, and/or (b) the metastatic lesion became significantly high signal on T2WI, no high signal on diffusion-weighted imaging (DWI), no low signal on apparent diffusion coefficient (ADC), and no enhancement after contrast administration. (Figure 2) The location and size of liver lesions were determined according to imaging manifestations, and the size was calculated according to the long and longitudinal axes of tumors.

### 2.3. Treatment Regimens

In this study, the treatment regimens included chemotherapy alone, targeted therapy alone, and chemotherapy combined with targeted therapy. The chemotherapy regimen included oxaliplatin-based chemotherapy and irinotecan-based chemotherapy. The targeted drugs were bevacizumab and cetuximab.

### 2.4. Definition and Follow-up

When CRLM, in initial diagnosis, was assessed that cannot guarantee enough liver parenchyma during surgery or technically completely resected, the lesions were defined as the initially unresectable liver metastasis lesions [19–21]. The liver metastasis not detected radiologically was defined as “disappearing liver metastasis” (DLM). The first diagnosis, occurrence, and recurrence time of the CRLM were defined as the time of initial diagnosis, occurrence, and recurrence.

The number of effective lesions in each period was calculated according to the total number of lesions, of lost lesions in the period, and of all recurrent and lost before the period. The lesion, not recurrent but lost to follow-up period or surgically removed during follow-up, was defined as the lost lesion.

Progression-free survival (PFS) time was defined as the time interval from the occurrence of a single DLM to recurrence. The follow-up time nodes were as follows: (a) the time of initial diagnosis of liver metastasis; (b) the occurrence time of each DLM; and (c) the time of recurrence of each DLM.

### 2.5. Data Collection

The clinical baseline information was retrospectively collected, including gender, age, site of primary tumor, body mass index (BMI), carcinoembryonic antigen (CEA), carbohydrate antigen 19-9 (CA19-9), simultaneous/metachronous liver metastasis, extrahepatic metastasis, conversion treatment regimens, and lymph node metastasis of primary tumor. The imaging information included the time of initial diagnosis, the size, number, and segment of liver metastasis. Each follow-up period information had follow-up time, the disappearance of liver metastasis lesions, disappearing time of the lesions, segment of the DLM, size of the DLM at initial diagnosis, number of DLM, recurrence of DLM, time of recurrence of the DLM, segment, and the number of recurrent DLM.

### 2.6. Statistical Analysis

SPSS 22.0 software was used for statistical analysis. Count data were compared by *χ*^2^ test. Progression-free survival (PFS) time was analyzed by the Kaplan-Meier method and Log-rank test, and multivariate analysis was by Cox proportional regression model. *P* < 0.05 was considered as a statistically significant difference.

## 3. Results

### 3.1. Baseline Characteristics of Patients with DLM

Three hundred forty-five patients were identified as CRC from January 2014 to January 2021. According to the inclusion and exclusion criteria, 113 patients with CRLM were included. According to the imaging data analysis, 35 patients (31.0%) had DLM in 113 patients, and there were more than 361 liver metastasis lesions in total. Of the 361 liver lesions, 177 lesions showed radiologic disappearance (49.0%), known as DLM. Two patients had too many liver metastases not to count accurately, so we excluded the number of liver lesions in these two patients, a total of 60, in which there were 32 DLM (Table 1). Of the 177 DLM, a total of 17 lesions were lost to follow-up after the initial diagnosis of disappearing lesions, and 6 liver metastases were resected in the subsequent surgical treatment.

### 3.2. The Correlation between Time and Recurrence of DLM

According to the time of the remaining disappeared status, the liver lesions were divided into five groups, including 6 months, 6-12 months, 12-24 months, 24-36 months, and 36 months to present. Among them, the number of the recurrence of DLM within 6 months was 5 (3.4%), 6-12 months group was 20 (16.8%), and 12-24 months group was 24 (34.8%), but of the 24-36 months group and 36 months to present group, there was no recurrence (Table 2); the difference among the groups was statistically significant (*P* < 0.001).

### 3.3. The Correlation between Treatment Regimens and Occurrence of DLM

The liver metastases were divided into three groups according to conversion therapy regimens, including chemotherapy alone group, targeted therapy alone group, and chemotherapy combined with the targeted therapy group. The difference among the three groups was statistically significant (*P* < 0.001) (Table 3). There was a statistical difference between the chemotherapy alone and chemotherapy combined with the targeted therapy groups (*P* < 0.001), while there was neither difference between the chemotherapy alone group and the targeted therapy alone group (*P* = 1.000) nor between the targeted therapy alone group and chemotherapy combined with the targeted therapy group (*P* = 0.268) (Table 4).

### 3.4. The Correlation of Segment and Size of Liver Metastasis Lesions and Occurrence of DLM

Groups were divided according to the segment and the size of liver metastases. The segments were divided into eight groups from S1-S8, and the size was divided into three groups: sizes <5 mm, 5-10 mm, and>10 mm, as shown in Table 3. There was no significant difference between the groups about their segment on the occurrence of DLM (*P* = 0.330). There were statistical differences among the three groups in the size of liver metastasis lesions on the occurrence of DLM (*P* < 0.001) (Table 3) in which there were statistical differences not only between <5 mm group and >10 mm group but also between 5-10 mm group and >10 mm group (*P* < 0.001); however, there was no statistical difference between the <5 mm group and 5-10 mm group (*P* = 0.478) (Table 4).

### 3.5. Univariate and Multivariate Analysis

**Figure 3:**
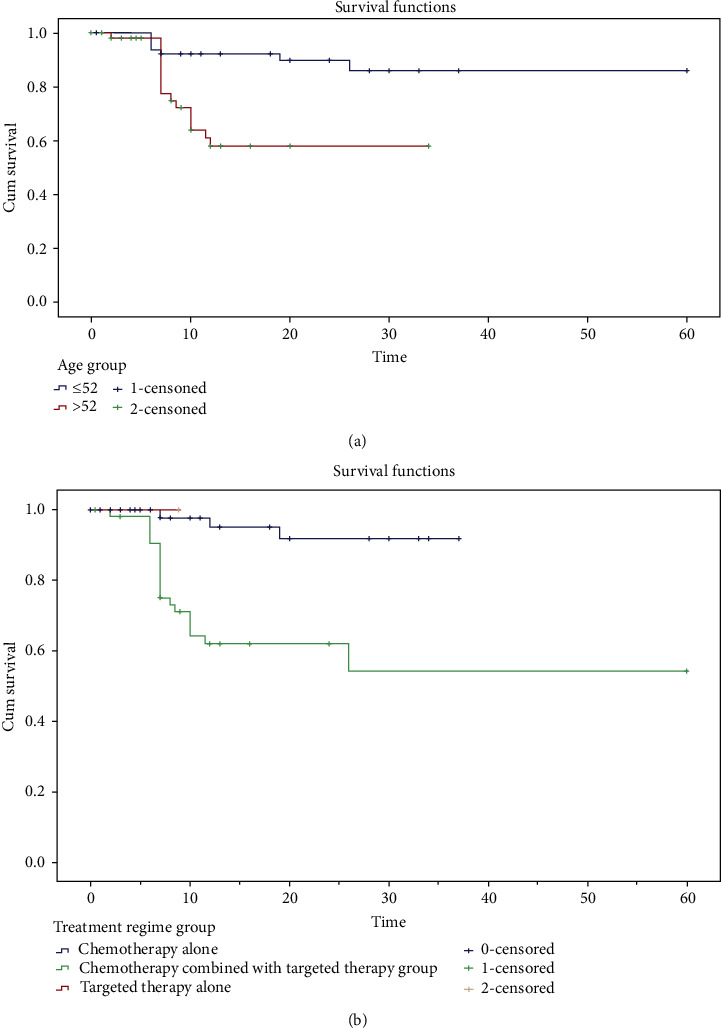
Progression-free survival (PFS) curves for patients with DLM. (a) A curve of PFS time related to age for DLM patients. (b) A curve of PFS time related to the treatment regimens for DLM patients. Abbreviations: DLM: disappearing liver metastasis; PFS: progression-free survival.

Sex, age, site of the primary tumor, BMI, CEA, CA19-9 at admission, simultaneous/metachronous liver metastasis, size of liver metastasis, extrahepatic metastasis, conversion treatment regimens, and lymph node metastasis of the primary tumor were analyzed and compared. Finally, age, sex, and treatment regimens were thought to influence the PFS time of DLM. After that, the relevant factors were incorporated into COX multivariate regression equation, and age and treatment regimens were analyzed as independent influencing factors (Table 5). Then, the PFS time curve between groups was made by K-M curve for the two independent factors. In the K-M curve analysis, the PFS time of DLM in the younger group (<52) was significantly higher than that in another group. The figure showed that the final tumor-free status of the younger group was higher than 80% (Figure 3). Among the different treatment regimens groups, it was obvious that the PFS time of the chemotherapy alone and the targeted therapy alone groups was significantly higher than that of chemotherapy combined with the targeted drugs group, and the tumor-free status of the first two groups was higher than 90% (Figure 3).

## 4. Discussion

Conversion therapy for initially unresectable liver metastasis of CRC patients has a significant role. Some studies have claimed that in the process of conversion therapy, some liver lesions of 9-37% patients with CRLM are not visible on the radiology, known as DLM [2, 6, 21–23]. This study shows DLM in 31.0% of 113 patients, similar to the previous studies.

In time groups, we can conclude that the recurrence rate of the DLM is rising year by year within two years, which is similar to some studies mentioning that recurrence rates of patients with DLM are higher in the two years [13]. But there is no tumor progression after two years, even a portion of the DLM at five years without recurrence. As the number of lesions is less in follow-up three years and five years in the study, the conclusion may be biased. We consider that if we can follow up to see the residual DLM on the liver and no tumor recurrence for 2 years, the lesions might be deemed complete pathological lesion disappearance.

Some studies have shown that oxaliplatin-based chemotherapy regimen is more prone to the occurrence of disappearing lesions [23]. In our research, the chemotherapy alone group is statistically significant with chemotherapy combined with the targeted therapy group, suggesting that patients with CRLM who can be treated with chemotherapy alone are more likely to have DLM. This conclusion is relatively broad than the conclusion of the previous studies. We think that this result may be caused by the differences in gene mutation in CRC patients' tumors or the different treatment cycles. And in this study, the targeted therapy alone group is not significant with other groups. We think it may be caused for the small number of lesions of this targeted therapy alone group.

The segment of liver metastasis does not correlate with the occurrence of DLM, but the size of liver lesions is correlated. Some studies have claimed that less than 20 mm liver lesions in the treatment process are more prone to make lesions disappear [16]. But our research suggests that <5 mm and 5-10 mm lesions are more prone to disappear lesions than >10 mm, which indicates that if the size of liver metastasis lesions in patients is less or equal to 10 mm, patients are more likely to develop DLM through conversion therapy.

Univariate analysis shows that gender, age, and different treatment regimens impact the PFS time of the disappearing liver metastasis lesions. The age and treatment regimens are independent factors in PFS time for patients through the COX multivariate regression equation. We can see from the K-M survival analysis curve that PFS time is higher in younger patients (<52) and treated with chemotherapy or targeted therapy alone.

There are some limitations of this study as well. First, it is a single-center retrospective study with 113 patients, which is relatively small. Second, due to the external factors of the patients themselves, they could not meet the 5-year follow-up in our study. Therefore, multicenter and large sample randomized controlled trials (RCTs) with patients with DLM are needed in the future experiments.

In conclusion, patients with CRLM who are younger (<52) and treated with chemotherapy have higher PFS time. And chemotherapy alone is more likely to have DLM for the patients. And if DLMs have no recurrence within 2 years, the lesions might be considered to have complete pathological disappearance. So in this study, we think that younger patients, who used chemotherapy alone to achieve a therapeutic effect, might have better survival benefits when the lesions do not progress within 2 years after the appearance of DLMs.

## Figures and Tables

**Table 1:** Baseline characteristics of patients corresponding single DLM.

Characteristics	No. 301
Sex	
Male	161 (53.5%)
Female	140 (46.5%)
Age (mean ± SD) (year)	52.5 ± 10.7
BMI preoperative (mean ± SD) (kg/m^2^)	23.4 ± 2.73
CEA preoperative (mean ± SD) (ng/ml)	294.8 ± 546.3
CA 19-9 preoperative (mean ± SD) (u/ml)	5227.8 ± 19084.2
Tumor site	
Colon	175 (58.1%)
Rectum	126 (41.9%)
Simultaneous/metachronous liver metastases	
Simultaneous	263 (87.4%)
Metachronous	38 (12.6%)
Extrahepatic metastasis	
Yes	136 (45.2%)
No	165 (54.8%)
Conversion therapy regimens	
Chemotherapy alone	162 (53.8%)
Targeted therapy alone	10 (3.3%)
Chemotherapy combined targeting	129 (42.9%)
Lymph node metastasis	
Yes	136 (45.2%)
No	66 (21.9%)
Data loss	99 (32.95%)
Site of liver metastases	
S1	6 (2.0%)
S2	24 (8.0%)
S3	25 (8.3%)
S4	34 (11.3%)
S5	40 (13.3%)
S6	41 (13.6%)
S7	57 (18.9%)
S8	74 (24.6%)
Size of liver metastases	
<5 mm	30 (10.0%)
5-10 mm	100 (33.2%)
>10 mm	171 (56.8%)

Note: Variables are expressed as the mean ± SD or *n* (%). Abbreviations: DLM: disappearing liver metastasis; BMI: body mass index; CEA: carcinoembryonic antigen; CA 19-9: carbohydrate antigen 199.

**Table 2:** The correlation of time nodes and DLM.

	The number of recurrent liver metastases	The number of liver metastases lost in follow-up	The number of liver metastases effectively disappeared	The number of no recurrent liver metastases	^∗^ *P* value
Time (months)					<0.001
6	5	13	147	142	
6-12	20	23	119	99	
12-24	24	30	69	45	
24-36	0	17	28	28	
36-present	0	21	7	7	

Note: ^∗^*P* value <0.05. Abbreviations: DLM: disappearing liver metastasis.

**Table 3:** The correlation of different factors and DLM.

	Disappearing	No disappearing	^∗^ *P* value
Conversion therapy regimens			<0.001
Chemotherapy alone	112	80	
Targeted therapy alone	6	4	
Chemotherapy combined targeting	59	100	
Site of liver metastases			0.330
S1	3	3	
S2	9	15	
S3	16	9	
S4	18	16	
S5	14	26	
S6	22	19	
S7	30	27	
S8	33	41	
Size of liver metastases			<0.001
<5 mm	23	7	
5-10 mm	70	30	
>10 mm	52	119	

Note: ^∗^*P* value <0.05. Abbreviations: DLM: disappearing liver metastasis.

**Table 4:** The correlation of different factors and DLM.

	Disappearing	No disappearing	^∗^ *P* value
Conversion therapy regimens			1.000
Chemotherapy alone	112	80	
Targeted therapy alone	6	4	
Conversion therapy regimens			<0.001
Chemotherapy alone	112	80	
Chemotherapy combined targeting	59	100	
Conversion therapy regimens			0.268
Targeted therapy alone	6	4	
Chemotherapy combined targeting	59	100	
Size of liver metastases			0.478
<5 mm	23	7	
5-10 mm	70	30	
Size of liver metastases			<0.001
<5 mm	23	7	
>10 mm	52	119	
Size of liver metastases			<0.001
5-10 mm	70	30	
>10 mm	52	119	

Note: ^∗^*P* value <0.05. Abbreviations: DLM: disappearing liver metastasis.

**Table 5:** Univariate and multivariate analysis of progression-free survival.

Risk factors	Univariate analysis		Multivariate analysis	
	HR (95% CI)	^∗^ *P* value	HR (95% CI)	^∗^ *P* value
Age (>/≤52, years)	4.532 (1.785-11.503)	0.001	3.690 (1.173-11.607)	0.026
Sex (male/female)	5.016 (1.753-14.349)	0.003	1.570 (0.430-5.736)	0.495
Size of liver metastasis (>/≤5 mm)	2.715 (0.806-9.143)	0.107		
Primary site (colon/rectum)	1.476 (0.628-3.469)	0.372		
Extrahepatic metastasis (yes/no)	1.002 (0.433-2.318)	0.996		
T staging of the primary tumor	0.398 (0.103-1.536)	1.181		
Lymph node metastasis (yes/no)	7.706 (0.936-63.390)	0.058		
BMI (>/≤23.4)	0.646 (0.270-1.544)	0.326		
CEA (>/≤5)	2.140 (0.889-5.152)	0.090		
CA19-9(>/≤40)	0.930 (0.377-2.294)	0.875		
Liver metastasis (simultaneous/metachronous)	1.441 (0.486-4.266)	0.510		
Treatment regimens (chemotherapy alone/targeted therapy alone/chemotherapy combined with targeted therapy group)	2.593 (1.310-5.134)	0.006	2.703 (1.081-6.758)	0.033
Segment of liver metastases	0.928 (0.744-1.157)	0.506		

Note: ^∗^*P* value <0.05. Abbreviations: PFS: progression-free survival; CI: confidence interval; BMI: body mass index; CEA: carcinoembryonic antigen; CA 19-9: carbohydrate antigen 199.

## Data Availability

The data of this study are available upon special request to the corresponding author.
